# Enhancement of lysine biosynthesis confers high-temperature stress tolerance to *Escherichia coli* cells

**DOI:** 10.1007/s00253-021-11519-0

**Published:** 2021-08-29

**Authors:** Shota Isogai, Hiroshi Takagi

**Affiliations:** Division of Biological Science, Graduate School of Science and Technology, Nara Institute of Science and Technology, 8916-5 Takayama-cho, Ikoma, Nara 630-0192 Japan

**Keywords:** *Escherichia coli*, Lysine, ThrA, Aspartate kinase, Homoserine dehydrogenase, Stress tolerance

## Abstract

**Abstract:**

Lysine, a nutritionally important amino acid, is involved in adaptation and tolerance to environmental stresses in various organisms. Previous studies reported that lysine accumulation occurs in response to stress and that lysine supplementation enhances stress tolerance; however, the effect of lysine biosynthesis enhancement on stress tolerance has yet to be elucidated. In this study, we confirmed that lysine supplementation to the culture medium increased intracellular lysine content and improved cell growth of *Escherichia coli* at high temperature (42.5 °C). Lysine-overproducing strains were then isolated from the lysine analogue *S*-adenosylmethionine-resistant mutants by conventional mutagenesis and exhibited higher tolerance to high-temperature stress than the wild-type strain. We identified novel amino acid substitutions Gly474Asp and Cys554Tyr on ThrA, a bifunctional aspartate kinase/homoserine dehydrogenase (AK/HSDH), in the lysine-overproducing mutants. Interestingly, the Gly474Asp and Cys554Tyr variants of ThrA induced lysine accumulation and conferred high-temperature stress tolerance to *E. coli* cells. Enzymatic analysis revealed that the Gly474Asp substitution in ThrA reduced HSDH activity, suggesting that the intracellular level of aspartate semialdehyde, which is a substrate for HSDH and an intermediate for lysine biosynthesis, is elevated by the loss of HSDH activity and converted to lysine in *E. coli*. The present study demonstrated that both lysine supplementation and lysine biosynthesis enhancement improved the high-temperature stress tolerance of *E. coli* cells. Our findings suggest that lysine-overproducing strains have the potential as stress-tolerant microorganisms and can be applied to robust host cells for microbial production of useful compounds.

**Key points:**

• *Lysine supplementation improved the growth of E. coli cells at high temperature.*

• *The G474D and C554Y variant ThrA increased lysine productivity in E. coli cells.*

• *The G474D substitution in ThrA reduced homoserine dehydrogenase activity.*

• *E. coli cells that overproduce lysine exhibited high-temperature stress tolerance.*

**Supplementary Information:**

The online version contains supplementary material available at 10.1007/s00253-021-11519-0.

## Introduction

Lysine, one of the essential amino acids for humans, also protects yeast cells from freezing, dehydration, and oxidative stresses (Takagi et al. 1997; López-Martínez et al. 2015; Olin-Sandoval et al. 2019). Plant cells accumulate lysine in response to multiple environmental stresses caused by drought (Du et al. 2015; Yadav et al. 2019; Demirel et al. 2020). In plants and animals, the genes encoding enzymes that degrade lysine to α-aminoadipate (AAA) via saccharopine are induced in response to osmotic and oxidative stress (Arruda and Barreto 2020). Some marine bacteria also carry the genes responsible for the degradation of lysine to AAA, and expression of these genes improved osmotic stress tolerance of *Escherichia coli* by the formation of a compatible solute, pipecolate (De Mello Serrano et al. 2012; Neshich et al. 2013).

On the other hand, a previous study in the yeast *Saccharomyces cerevisiae* reported that the addition of lysine to the culture medium switched the flux of NADPH, which is required for lysine biosynthesis, to the production of glutathione. The resulting increase in intracellular glutathione enhanced tolerance to oxidative stress (Olin-Sandoval et al. 2019). *E. coli* cells maintain pH homeostasis to form cadaverine by decarboxylation of lysine with intracellular protons and exchange external lysine for internal cadaverine via a cadaverine-lysine antiporter (Kanjee and Houry 2013). These findings demonstrated that lysine is closely related to adaptation and tolerance to environmental stresses; however, the detailed mechanism of lysine-mediated stress tolerance in microorganisms has yet to be elucidated. Moreover, little is known about the effect of the enhancement of lysine biosynthesis on stress tolerance. Lysine-mediated stress tolerance could contribute to the construction of robust host strains for microbial production of useful compounds.

**Fig. 1:**
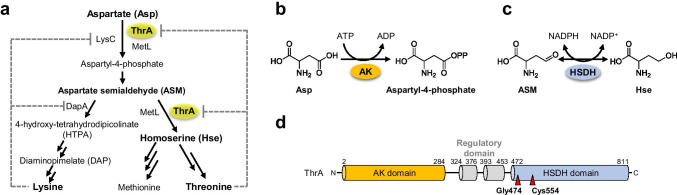
Biosynthesis of lysine in *E. coli* and characteristics of the ThrA protein. **a** Biosynthetic pathways of aspartate-derived amino acids (lysine, threonine, and methionine) in *E. coli*. Aspartate (Asp) is phosphorylated to aspartate-4-phosphate by aspartate kinase (LysC, ThrA, and MetL). Aspartate-4-phosphate is then converted into aspartate semialdehyde (ASM). In the lysine biosynthesis, 4-hydroxy-tetrahydrodipicolinate (HTPA) synthase catalyzes the conversion of ASM to HTPA. On the other hand, homoserine dehydrogenase (HSDH) catalyzes the reduction of ASM to homoserine (Hse) in the biosynthesis of threonine and methionine. The enzymatic activity of LysC and DapA are subjected to feedback inhibition by lysine, whereas that of ThrA is inhibited by threonine. LysC, aspartate kinase; DapA, 4-hydroxy-tetrahydrodipicolinate synthase; ThrA and MetL, bifunctional aspartate kinase/homoserine dehydrogenase. **b** The enzymatic reaction of AK. AK phosphorylates the carboxyl group in the side chain of Asp using adenosine triphosphate (ATP) to form aspartate-4-phosphate. **c** The reaction of HSDH. HSDH catalyzed the reduction of ASM to Hse and the oxidation of Hse to ASM using NADPH and NADP^+^ as redox partners, respectively. **d** Domain organization of ThrA. AK catalytic domain, two regulatory domains, and HSDH catalytic domain are represented as orange, gray, and light blue bars, respectively. Amino acid residues (Gly474 and Cys554) substituted in the lysine-overproducing strains, AEC28 and AEC106, are represented as red triangles

Most bacteria and plants biosynthesize lysine from aspartate via diaminopimelate (DAP) that is known as the DAP pathway (Fig. 1a) (Chatterjee et al. 1994; Scapin and Blanchard 1998). The DAP pathway consists of nine enzyme-catalyzed steps for lysine biosynthesis. Aspartate kinase (AK) catalyzes the first reaction of the DAP pathway to form aspartate-4-phosphate by the phosphorylation of aspartate using adenosine triphosphate (ATP) (Fig. 1b). Aspartate-4-phosphate is subsequently converted to aspartate semialdehyde (ASM), a common intermediate for the biosynthesis of lysine, threonine, and methionine (Fig. 1a). For lysine biosynthesis, 4-hydroxy-tetrahydrodipicolinate (HTPA) synthase converts ASM to HTPA, while homoserine dehydrogenase (HSDH) catalyzes the reduction of ASM to homoserine in the biosynthesis of threonine and methionine (Fig. 1c). Previous studies reported that AK, HTPA synthase, and HSDH are subject to feedback inhibition by the end products (Scapin and Blanchard 1998; Park and Lee 2010).

In *E. coli*, there are three isozymes of AK regulated in different manners (Umbarger 1978; Chassagnole et al. 2001; Viola 2001). AK-I, which is fused with HSDH as bifunctional AK/HSDH I, is encoded by the *thrA* gene, and the enzymatic activity of ThrA is inhibited by threonine; meanwhile, AK-II, which is also incorporated to HSDH (AK/HSDH II), is encoded by the *metL* gene, and the expression of *metL* is repressed by methionine. Finally, the third AK, AK-III, is the *lysC* gene product. LysC is monofunctional AK, and its AK activity is tightly regulated via feedback inhibition by lysine. Amino acid substitutions of LysC and ThrA for the removal of feedback inhibition by lysine and threonine, respectively, were identified and utilized for threonine production in *E. coli* (Ogawa-Miyata et al. 2001; Lee et al. 2003). Biochemical analysis of threonine-sensitive AK/HSDH from *Arabidopsis thaliana* revealed that two glutamine residues in the regulatory domains are important for threonine recognition (Paris et al. 2003). The domain organization of ThrA is predicted to be similar to that of *A. thaliana* AK/HSDH; the N-terminal AK and C-terminal HSDH catalytic domains with two small regulatory domains are located between these catalytic domains (Fig. 1d). These glutamine residues are conserved in ThrA, suggesting that the regulation of ThrA activity by threonine is similar to that of the *A. thaliana* enzyme. Although the *thrA* gene has been subjected to metabolic engineering for threonine production in *E. coli*, few studies have reported the engineering of the *thrA* gene for lysine production (Dong et al. 2011).

The present study found that the accumulation of intracellular lysine improved the high-temperature stress tolerance of *E. coli* cells. We identified novel amino acid substitutions, Gly474Asp and Cys554Tyr, on ThrA in the lysine-overproducing mutants. Enzymatic analysis of the G474D variant revealed that HSDH activity was reduced by glycine-to-aspartate replacement, leading to high production of lysine and enhanced tolerance to high-temperature stress in *E. coli* cells.

## Materials and methods

### Strains and culture media

An *Escherichia coli* strain BW38029 (F^−^ ∆(*araD-araB*)*567 lacZp4105*(*UV5*)*-lacY λ*^−^ *hsdR514*, an independent isolate of strain BW38028 (Conway et al. 2014) was kindly provided by Dr. Hirotada Mori (Division of Biological Science, Nara Institute of Science and Technology) and was used as a parental strain for *S*-adenosylmethionine (AEC)-resistant mutants and a host strain for expressing the ThrA variants. BW38029-derived strains were cultured in M9 medium (4 g/L glucose, 65 mM sodium/potassium phosphate, 8.6 mM NaCl, 18.7 mM ammonium chloride, and 1 mM MgSO_4_), unless otherwise stated. *E. coli* strains DH5α (F^−^ λ^−^ Φ*80lacZ*∆*M15* ∆(*lacZYA argF*)*U169 deoR recA1 endA1 hsdR17*(*r*_*k*_^−^*m*_*k*_^+^) *supE44 thi-1 gyrA96*) and BL21 (DE3) (F^−^ *ompT hsdS*(r_B_^−^ m_B_^−^) *gal dcm λ*(DE3) (λ(DE3):*lacI*, *lacUV5-T7 gene1 ind1 sam7 nin5*) were used for construction of expression plasmids and for expression of the recombinant ThrA, respectively. These *E. coli* strains were cultured in Luria–Bertani (LB) medium (5 g/L yeast extract, 10 g/L tryptone, and 5 g/L NaCl) containing appropriate antibiotics or in M9CA medium (M9 medium supplemented with 20 g/L casamino acid) containing 100 μg per mL ampicillin.

### Isolation of lysine analogue-resistant mutants

The wild-type (WT) strain BW38029 was randomly mutagenized by treatment with 2% of ethyl methanesulfonate (EMS) in phosphate-buffered saline (PBS; pH7.4) at 37 °C for 45 min. Mutagenized cells were washed with 10% (w/w) sodium thiosulfate in PBS twice and then suspended in PBS. Approximately 5 × 10^6^ cells were spread onto an M9 medium containing 100 μg per mL of AEC. After being cultivated at 37 °C for 2 days, the resulting colonies were collected, and then AEC resistance and lysine production were analyzed.

### AEC sensitivity of *E. coli* cells

BW38029-derived strains were pre-cultured at 37 °C. After 24 h of cultivation, *E. coli* cells were harvested, washed by PBS twice, and suspended in PBS. The suspension was serially diluted to an optical density at 600 nm (OD_600_) of 10^−1^ to 10^−5^, then spotted onto M9 agar medium without or with 100 μg/mL of AEC, and incubated at 37 °C for 24 h.

### Measurement of intracellular amino acids contents

BW38029-derived strains were pre-cultivated at 37 °C overnight and then inoculated to a new medium at an OD_600_ of 0.05. After cultivation at 37 °C for 24 h, *E. coli* cells were collected by centrifugation and washed twice with sterilized water. Harvested cells were resuspended in sterilized water, and the suspension was adjusted to an OD_600_ of 40. Consequently, intracellular amino acids in an aliquot (0.15 mL) of the cell suspension were extracted by boiling at 100 °C for 10 min. Cell debris was removed by centrifugation, and each supernatant was subsequently quantified with an UF-amino station (Shimadzu, Kyoto, Japan) with pre-column derivatization using 3-aminopyridyl-N-hydroxysuccinimidyl carbamate (Wako Pure Chemical, Osaka, Japan). The content of each amino acid was represented as μmol per gram dry cell weight (DCW).

### Whole genome sequence analysis

The extracted genomic DNAs from the parent strain (BW38029) and the AEC-resistant strain (AEC28) were quantified with Qubit (Thermo Fisher Scientific, Waltham, MA). A next-generation sequencing library was constructed for each genome using the Nextera DNA Library Preparation Kit (Illumina, San Diego, CA) according to the manufacturer’s instructions. The genome libraries were sequenced using MiSeq (Illumina) with MiSeq Reagent Kit v2 or v3 (Illumina). Sequencing data processing of BW38029 and AEC28, as well as sequencing data from the Sequence Read Archive (SRA), was performed with CLC Genomics Workbench v 10.1.1 (Qiagen, Hilden, Germany). This process included trimming, mapping, and variants calling against the reference genome of *E. coli* BW25113 (GCA_000750555). Reads bases not matching in the alignment were scored as variants. The coverage table files and the variants table files were exported from Genomics Workbench and retained for further analysis. These files were converted into a FASTA file of synthetic sequences with custom scripts. These scripts generate the sequences of homozygous SNPs from the data of coverage and variants. The sequencing data were deposited to DNA Data Bank of Japan (DDBJ) sequence read archive (DRA). The accession numbers of strains BW38029 and AEC28 are SAMD00324911 and SAMD00324912, respectively.

### Construction of *E. coli* strains expressing the ThrA variants

To construct strains expressing the G474D and C554Y ThrA variants, an open reading frame (ORF) of *thrA* in the genomic DNA was replaced with the mutant genes, *thrA*^G474D^ and *thrA*^C554Y^ (corresponding to G474D and C554Y substitutions, respectively), using λ-red recombination system (Datsenko and Wanner 2000). The *thrA*^G474D^ and *thrA*^C554Y^ genes were amplified using the genomic DNA of AEC28 and AEC106 as templates with primers thrA_in_fusion_fw (5′-TCG AAT TCA AAG GAG GTA CCC ACC ATG CGA GTG TTG AAG TTC GG-3′) and rv (5′-GAG ACA ACT TCT AGA TCA GAC TCC TAA CTT CCA TGA GAG GG-3′). The amplified DNA fragments were sub-cloned into pSF-OXB1 vector (OXGENE, Oxford, UK) using by In-Fusion HD Cloning Kit (Takara Bio, Shiga, Japan). After the nucleotide sequences were verified, the DNA fragments including the *thrA* ORF were obtained by digesting with *Bam*HI and *Eco*RI and then ligated into the same sites of pK18mobSacB vector (Kvitko and Collmer 2011) resulted in pKMS_*thrA*^*G474D*^ and pKMS_*thrA*^*C554Y*^. These plasmids were linearized by digestion of *Ssp*I for pKMS_*thrA*^*G474D*^ and *Mun*I for pKMS_*thrA*^*C554Y*^, respectively. BW38029 harboring pKD46 (Datsenko and Wanner 2000) was cultured in LB medium including arabinose to induce the gene expression of λ-red recombinase and then transformed with the aforementioned linearized DNA. Single crossover strains, which harbor the full length of pKMS_*thrA*^*G474D*^ and pKMS_*thrA*^*C554Y*^ in the *thrA* locus by homologous recombination, were selected by growth phenotype (kanamycin^r^ and sucrose^s^) and confirmed by PCR analysis. To induce second-time recombination, single-crossover strains were grown to an OD_600_ of 0.8, and then harvested cells were spread onto LB medium (without NaCl) containing 100 g/L sucrose. Double crossover strains were picked up by growth phenotype (kanamycin^s^ and sucrose^r^), and objective strains, which harbor *thrA*^G474D^ and *thrA*^C554Y^ in the *thrA* locus, were selected by PCR analysis from double crossover strains (G474D and C554Y, respectively). Replacement of the *thrA* ORF to *thrA*^G474D^ and *thrA*^C554Y^ was confirmed by DNA sequencing.

### Construction of plasmids for expressing the recombinant ThrA

To construct plasmids for expressing the recombinant ThrA enzymes, the WT and the mutant *thrA* genes were amplified from the genomic DNA of WT, AEC28, and AEC106 by PCR with the primers thrA_gateway_Fw (5′-GGG GAC AAG TTT GTA CAA AAA AGC AGG CTT AAT GCG AGT GTT GAA GTT CGG-3′) and Rv (5′-GGG GAC CAC TTT GTA CAA GAA AGC TGG GTG GAC TCC TAA CTT CCA TGA GAG G-3′). The PCR-amplified DNA fragment was introduced into the pDONR221 vector (Thermo Scientific, Waltham, MA) using BP clonase II (Thermo Scientific, Waltham, MA), resulting in pDONR221_*thrA*, pDONR221_*thrA*^*G474D*^, and pDONR221_*thrA*^*C554Y*^. The nucleotide sequences of the *thrA* genes were verified, and they were transferred to the pET53-dest expression vector (Thermo Scientific, Waltham, MA) using LR clonase II (Thermo Scientific, Waltham, MA), resulting in pET53_*thrA*, pET53_*thrA*^*G474D*^, and pET53_*thrA*^*C554Y*^.

### Expression and purification of the recombinant ThrA

*E. coli* BL21 (DE3) cells harboring pET53_*thrA*, pET53_*thrA*^*G474D*^, and pET53_*thrA*^*C554Y*^ were cultivated in M9CA medium containing ampicillin and grown at 37 °C to an OD_600_ of 0.8. The cells were cooled on ice for 5 min, and isopropyl β-d-1-thiogalactopyranoside (IPTG) was added to a final concentration of 0.2 mM. After 20 h of cultivation at 18 °C, the cells were harvested by centrifugation and suspended in buffer A (50 mM Tris–HCl (pH 7.4), 500 mM NaCl, and 20% (w/w) glycerol). The cell suspension was homogenized under cooling and then centrifuged to remove the insoluble fraction. The supernatant was filtrated by a 0.45-μm filter and subsequently applied onto a nickel affinity column (Ni Sepharose™ 6 Fast flow, GE Healthcare Life Sciences, Chicago, IL). After the column was washed with buffer A containing 40 mM imidazole, the recombinant proteins were eluted by buffer A supplemented with 500 mM imidazole. The elution fraction was dialyzed twice with buffer containing 50 mM Tris–HCl (pH 7.4), 150 mM NaCl, and 10% (w/w) glycerol at 4 °C. Proteins were quantified using Bio-Rad Protein Assay (Bio-Rad, Hercules, CA) and subjected to SDS–polyacrylamide gel electrophoresis.

### Enzymatic analysis of the recombinant ThrA

AK activity was measured by the production of ADP in an enzyme-coupled system with pyruvate kinase (PK) and lactate dehydrogenase (LDH) (Wampler and Westhead 1968; Chassagnole et al. 2001; James and Viola 2002). The reaction mixture (final volume, 1 mL) contained the following: 100 mM HEPES–NaOH (pH7.5), 100 mM KCl, 10 mM MgCl_2_, 1 mM phosphoenolpyruvate, 0.25 mM NADH, 15 U of PK/LDH (Sigma-Aldrich, St. Louis, MO), 2 μg of purified ThrA, and various concentrations of aspartate and ATP. The reaction mixture except for aspartate was pre-equilibrated for 3 min at 37 °C, and then the reaction was initiated by the addition of aspartate. ThrA-dependent oxidation of NADH was monitored at 340 nm with a DU-800 spectrophotometer (Beckman Coulter, Brea, CA) and maintained at 37 °C. For steady-state kinetics, when the concentration of aspartate was kept at 10 mM, the concentrations of ATP were varied (0.5–10 mM). With a fixed concentration of 10 mM ATP, the concentration of aspartate was 0.5–10 mM. In order to examine the feedback inhibition sensitivity of ThrA, the concentration of aspartate and ATP was fixed at 6 and 10 mM, respectively, and threonine was added to the reaction mixture at a concentration of 0.05–1 mM. The reaction rate was calculated with the extinction coefficient of NADH, 6220 M^−1^‧cm^−1^. One unit of activity was defined as the amount of enzyme required to produce 1 μmol of ADP per min.

HSDH activity (reverse direction) was measured by the synthesis of NADPH. The reaction mixture (final volume, 1 mL) contained 100 mM HEPES–NaOH (pH7.5), 100 mM KCl, 5 mM NADP^+^, 30 μg of purified ThrA, and 1–5 mM of homoserine. The reaction mixture except for homoserine was pre-equilibrated for 3 min at 37 °C, and then the reaction was initiated by the addition of homoserine. ThrA dependent reduction of NADP^+^ was monitored at 340 nm with a DU-800 spectrophotometer and maintained at 37 °C. The reaction rate was calculated with the extinction coefficient of NADPH, 6220 M^−1^‧cm^−1^. One unit of activity was defined as the amount of enzyme required to produce 1 μmol of NADPH per min. Kinetic parameters of each enzyme were calculated with GraphPad Prism version 9 (GraphPad Software, San Diego, CA) using nonlinear regression analysis.

## Results

### Enhancement of high-temperature stress tolerance of *E. coli* by intracellular lysine accumulation

**Fig. 2:**
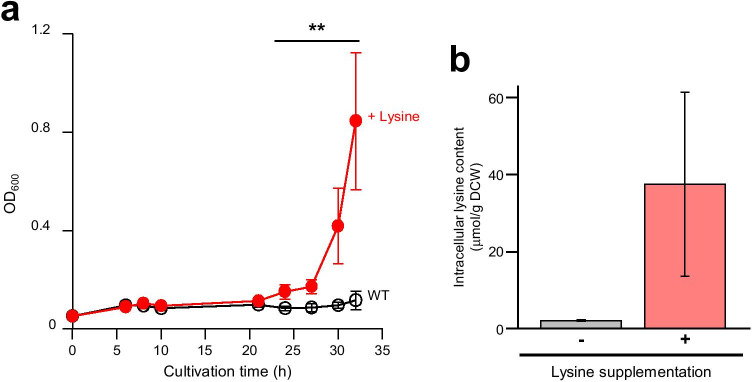
Effect of lysine supplementation on the growth of *E. coli* cells under high-temperature cultivation. **a** Growth curve of the WT strain BW38029 cultivated at 42.5 °C with or without 15 mM of lysine supplementation. Open black and filled red circles indicate without and supplemented with 15 mM of lysine, respectively. Supplementation of 15 mM lysine significantly improved the growth after cultivation for 24 h (indicated by asterisks). **b** Intracellular lysine contents of the WT strain BW38029 in M9 medium without (a gray bar) and with (a red bar) 10 mM of lysine supplementation. Intracellular lysine contents after 24 h cultivation were represented as micromoles per gram dry cell wright (DCW). Asterisks indicate statistically significant differences between two strains (Student’s *t*-test, **p < 0.01)

To confirm that intracellular lysine accumulation confers stress tolerance to *E. coli* cells, we first examined the effect of lysine supplementation to the growth medium on *E. coli* cells under high-temperature culture conditions (Fig. 2a). When the WT strain was cultivated in M9 medium at 42.5 °C, the *E. coli* cells could hardly grow. In contrast, supplementation of 15 mM lysine significantly improved growth after cultivation for 24 h even at 42.5 °C. When WT cells were cultivated in an M9 medium containing 10 mM lysine, intracellular lysine content was about 5- to 25-folds higher than that under cultivation in M9 medium without lysine supplementation (Fig. 2b). These results suggest that the addition of lysine to the culture medium increased intracellular lysine, leading to high-temperature stress tolerance of *E. coli*. It was therefore expected that *E. coli* cells that overproduce lysine would exhibit stress tolerance due to the intracellular lysine accumulation.

### Isolation of *E. coli* mutants with lysine accumulation from AEC-resistant mutants

**Fig. 3:**
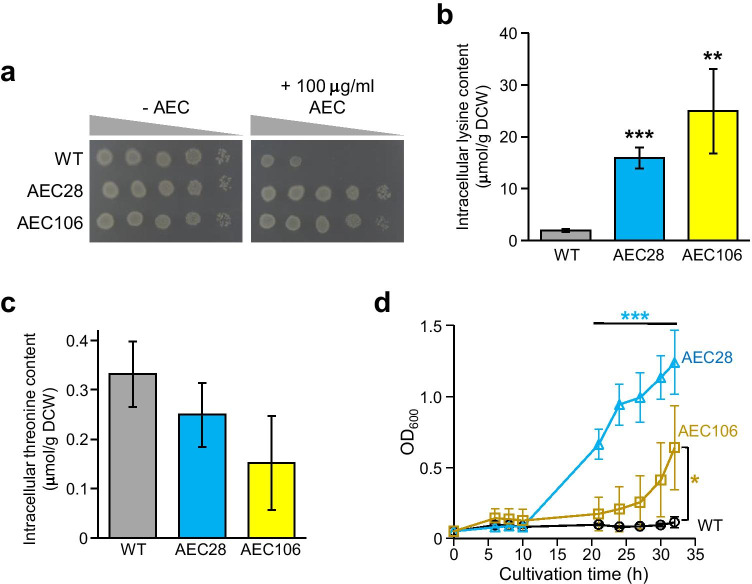
Phenotypes of AEC-resistant mutants. **a** AEC sensitivity of strains WT and AEC-resistant mutants (AEC28 and AEC106). Each strain was serially diluted to 10^1^- to 10^4^-folds (from left to right) and spotted onto M9 agar medium without (left panel) or with 100 μg/mL of AEC (right panel). Intracellular lysine (**b**) and threonine (**c**) contents of strains WT and AEC-resistant mutants. Intracellular amino acids contents after 24 h of cultivation in M9 medium were represented as micromoles per gram DCW. **d** Growth curve of strains WT and AEC-resistant mutants cultivated at 42.5 °C. Open black circles, open blue triangles, and open orange squares represent strains WT, AEC28, and AEC106, respectively. Asterisks indicate statistically significant differences between two strains (Student’s *t*-test, *p < 0.05, **p < 0.01, ***p < 0.001)

To obtain lysine high producers with enhanced high-temperature stress tolerance, we isolated the lysine toxic analogue AEC-resistant mutants, AEC28 and AEC106, by conventional mutagenesis (Fig. 3a). When these two mutants were cultured in M9 medium, intracellular lysine content was dramatically increased 8.2- and 12-folds, respectively, compared to that of the WT strain (Fig. 3b). On the other hand, it appears that the intracellular threonine contents in these mutants were decreased compared with that of the WT strain (Fig. 3c). Interestingly, we found that cell growth in both AEC28 and AEC106 mutants at 42.5 °C was greatly improved compared to that of the WT strain (Fig. 3d), which is similar to the case of lysine supplementation (Fig. 2a). These results indicate that the intracellular lysine content conferred high-temperature stress tolerance to *E. coli* cells.

### Identification of mutations on the *thrA* gene of lysine-overproducing mutants

Next, in order to identify the mutation(s) that induced lysine accumulation in *E. coli* cells, we conducted next-generation sequencing for the whole genome of strain AEC28. We identified a novel mutation in the *thrA* gene, which encodes the bifunctional AK/HSDH, corresponding to one amino acid replacement of Gly to Asp at position 474. In addition, a nucleotide sequence analysis revealed that strain AEC106 also carried a new mutation in the *thrA* gene, which led to a single amino acid substitution of Cys with Tyr at position 554. On the other hand, strains AEC28 and AEC106 had no mutations in the *lysC* and *dapA* genes, which encode the lysine feedback-inhibition sensitive AK and HTPA synthase, respectively. These results suggest that the two novel amino acid substitutions in the ThrA protein (Gly474Asp and Cys554Tyr) induced lysine accumulation in *E. coli* cells.

### Effects of the *thrA* gene mutation on lysine productivity and high-temperature stress tolerance

**Fig. 4:**
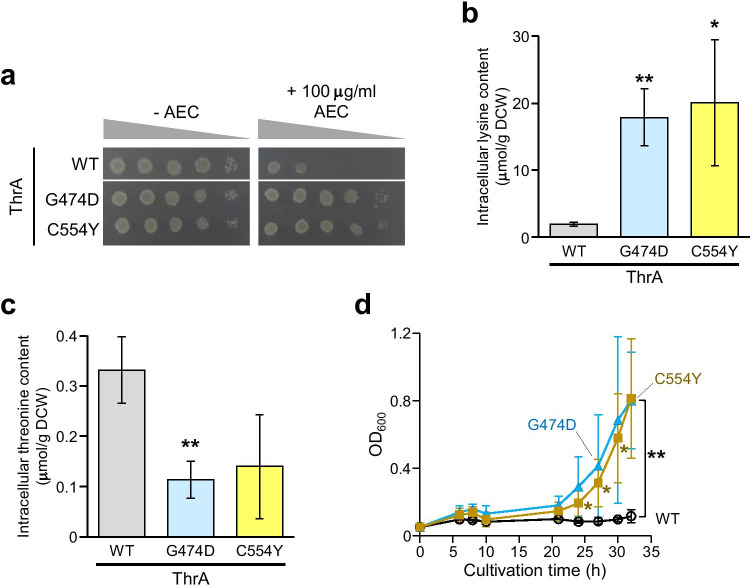
Phenotypes of *E. coli* expressing the ThrA variants. The *thrA* locus in the genome of *E. coli* was replaced with the mutant genes, *thrA*^G474D^ and *thrA*^C554Y^, resulting in strains G474D and C554Y. **a** AEC sensitivity of strains WT, G474D, and C554Y. Each strain was serially diluted to 10^1^- to 10^4^-folds (from left to right) and spotted onto M9 agar medium without (left panel) or with 100 μg/mL of AEC (right panel). Intracellular lysine (**b**) and threonine (**c**) contents of strains WT, G474D, and C54Y. Intracellular amino acids contents after 24 h of cultivation in M9 medium were represented as micromoles per gram DCW. **d** Growth curve of strains WT, G474D, and C554Y cultivated at 42.5 °C. Open black circles, filled blue triangles, and filled orange squares represent strains WT, G474D, and C554Y, respectively. Asterisks indicate statistically significant differences between two strains (Student’s *t*-test, *p < 0.05, **p < 0.01)

To analyze the effects of the *thrA* gene mutation on lysine productivity and high-temperature stress tolerance of *E. coli* cells, the WT *thrA* gene on the genome was replaced with its mutant genes (*thrA*^G474D^ and *thrA*^C554Y^). As shown in Fig. 4a, the WT strain carrying the *thrA* gene was sensitive to AEC, but *E. coli* cells harboring the *thrA*^G474D^ and *thrA*^C554Y^ genes (represented as strains G474D and C554Y, respectively) showed greatly increased resistance to AEC, suggesting that these ThrA variants enhanced lysine productivity in *E. coli* cells.

Next, we determined the cellular amino acid levels of *E. coli* strains (Fig. 4b, c). When *E. coli* strains were cultivated in M9 medium, a small amount of lysine and threonine was detected in the WT strain. In contrast, the lysine contents in strains G474D and C554Y were increased to 9.2- and 10.3-folds that of the WT strain, respectively, whereas strains G474D and C554Y exhibited lower threonine levels than the WT strain. These amino acid profiles in strains G474D and C554Y were almost the same as in the AEC28 and AEC106 mutants. We also monitored the cell growth of *E. coli* strains under high-temperature conditions (Fig. 4d). Interestingly, the mutant *thrA* genes (*thrA*^G474D^ and *thrA*^C554Y^) significantly reversed the growth inhibition of the WT strain at 42.5 °C. These results indicate that the *thrA*^G474D^ and *thrA*^C554Y^ genes induced lysine accumulation in *E. coli* cells. Moreover, the intracellular lysine enhanced the high-temperature stress tolerance of *E. coli*.

### Effects of amino acid substitutions on the enzymatic activities of ThrA

To further analyze the influence of amino acid substitutions at positions 474 and 554 on the enzymatic activities of ThrA, we attempted to express and purify the recombinant ThrA proteins using *E. coli* cells. Unfortunately, probably due to the insolubilization of the recombinant proteins in the cell, the C554Y variant ThrA did not exhibit detectable activity, suggesting that this variant could not express full activity (data not shown). On the other hand, the recombinant WT and G474D variant ThrA were obtained as soluble proteins. We purified these proteins to give a single band on SDS–polyacrylamide gel electrophoresis (Fig. S1) and evaluated their enzymatic properties for AK activity. As shown in Table 1, there were no significant differences in the kinetic constants (the *K*_m_ and *k*_cat_ values) or the *k*_cat_/*K*_m_ ratio between the WT and G474D variant enzymes. We also found that the AK activities of the WT and G474D variant ThrA in the presence of 0.5 mM threonine were 58% and 56%, respectively, of the activity in the absence of threonine. These results indicate that the Gly474Asp substitution in ThrA did not affect the catalytic activity or threonine-feedback inhibition sensitivity of AK.

**Table 1:** Kinetic parameters of the wild-type and variant ThrA

ThrA	AK activity (forward)						HSDH activity (reverse)		
	Aspartate			ATP			Homoserine		
	*K*_m_	*k*_cat_	*k*_cat_/*K*_m_	*K*_m_	*k*_cat_	*k*_cat_/*K*_m_	*K*_m_	*k*_cat_	*k*_cat_/*K*_m_
	(mM)	(s^−1^)	(s^−1^·mM^−1^)	(mM)	(s^−1^)	(s^−1^·mM^−1^)	(mM)	(s^−1^)	(s^−1^·mM^−1^)
WT	2.37 ± 0.47	16.1 ± 1.06	6.81	2.47 ± 0.39	17.3 ± 0.92	6.99	0.98 ± 0.48	0.022 ± 0.0028	0.022
G474D	3.21 ± 0.43	19.1 ± 0.96	5.95	1.95 ± 0.21	15.7 ± 0.52	8.03	ND^a^		

The values are the means and standard errors of results from three independent experiments. ^a^ND, not detected

Next, we attempted to measure the activity of HSDH, which catalyzes the reduction of ASM to homoserine with NADPH (the forward reaction). However, the ASM reduction activity could not be detected probably due to the instability of ASM. HSDH can also oxidize homoserine to ASM with NADP^+^ (the reverse reaction); thus, we evaluated the reverse activity (from homoserine to ASM) of the WT and G474D variant enzymes. The WT enzyme exhibited HSDH activity; Table 1 shows the *K*_m_ and *k*_cat_ values for homoserine in the WT enzyme. On the other hand, the HSDH activity of the G474D variant was not detected under the same conditions. These in vitro results indicate that the glycine-to-aspartate substitution at position 474 significantly reduced the HSDH activity. The loss of the HSDH activity could increase the intracellular level of ASM, which is the substrate of HSDH, and also an intermediate of lysine biosynthesis. Therefore, in *E. coli* cells that express the G474D variant ThrA, it is possible that the elevated ASM is preferentially converted into lysine, leading to lysine accumulation.

## Discussion

In this study, the intracellular lysine level was quite similar between AEC-resistant mutants (AEC28 and AEC106) and *E. coli* strains expressing the mutant *thrA* genes (G474D and C554Y). Our results indicate that both Gly474Asp and Cys554Tyr substitutions in ThrA are responsible for lysine hyper-production. The difference in growth improvement between strains AEC28 and G474D may be due to other mutation(s), which confer high-temperature stress tolerance, in strain AEC28. In this study, we showed that elevated lysine content conferred the high-temperature stress tolerance to *E. coli* cells; however, lysine supplementation to the culture medium also improved the growth of *E. coli* cells at low pH (Vivijs et al. 2016). Thus, in a future study, it is intriguing to investigate the effect of lysine accumulation on the growth phenotype under other stress conditions and to elucidate the molecular mechanism underlying the lysine-mediated stress tolerance.

In the yeast *S. cerevisiae*, the addition of lysine to the medium contributed to an increase in the intracellular NADPH pool and glutathione production, leading to enhancement of oxidative stress tolerance (Olin-Sandoval et al. 2019). Although lysine biosynthesis of *E. coli*, such as that of *S. cerevisiae*, requires redox cofactors, high production of lysine conferred high-temperature stress tolerance to *E. coli* cells as effectively as lysine supplementation. Therefore, the mechanism underlying the lysine-induced improvement of stress tolerance in *E. coli* may differ from that in *S. cerevisiae*. Our results suggest that biosynthesized lysine or its catabolites would protect the cellular components from damage caused by high temperature in *E. coli* in contrast to switching the flux of NADPH by lysine supplementation in *S. cerevisiae*. In particular, this possibility implies that lysine-overproducing microbes are expected to exhibit higher tolerance to environmental stresses and contribute to the construction of robust host strains for microbial fermentation. Arginine and glutamate have been reported to improve the stability and solubility of proteins (Golovanov et al. 2004). Like arginine and glutamate, lysine is a charged amino acid; thus, lysine accumulation may contribute to the prevention of the protein denaturation caused by high-temperature stress. Lysine functions as an ion-coating on the surface of membrane components and proteins in order to prevent denaturation by the NH_2_ groups in the molecule. In our preliminary experiments with the *S. cerevisiae* strain that accumulated intracellular lysine, when a precursor of lysine was added to the liquid medium, there was a significant increase in cell viability after freezing in water (Takagi et al. 1997). Furthermore, lysine is a kosmotropic compound due to the ammonium group in the side chain. Both high temperature and chaotropic compounds, such as organic solvents, urea, and guanidine, cause entropically disorder biomacromolecules. Previous studies reported that kosmotropes and compatible solutes confer to the stabilization of macromolecules caused by environmental stresses (Cray et al. 2015). For instance, supplementation of glycerol, one of the compatible solutes, to culture medium increased intracellular glycerol accumulation and enhanced benzene tolerance of *Pseudomonas putida* (Bhaganna et al. 2016). Therefore, the kosmotropic property of lysine may contribute to the protection of cells from the disordering of cellular macromolecules caused by high temperature similar to the protective effect of compatible solutes against chaotropic stress. Another possibility is that catabolite(s) of lysine have unknown functions to protect cells from high-temperature stress. For instance, some marine bacteria, plants, and animals induce the genes involved in the saccharopine pathway in response to environmental stresses for the degradation of lysine to AAA (Arruda and Barreto 2020). Although *E. coli* cells catabolize lysine via cadaverine and do not carry the genes responsible for the degradation of lysine to AAA, cadaverine confers stress tolerance to bacterial and plant cells (Kang et al. 2007; Rajpal and Tomar 2020).

In this study, the enzymatic properties of the C554Y variant ThrA could not be further characterized because we failed to prepare its recombinant enzyme. However, Cys554 is located in the HSDH domain such as Gly474; therefore, it is unlikely that the substitution of Cys to Tyr at position 554 affects the sensitivity to feedback inhibition by threonine. Furthermore, the intracellular contents of lysine and threonine in strain C554Y were similar to those of strain G474D. These results suggest that the cysteine-to-tyrosine substitution at position 575 would affect the HSDH activity of ThrA much like the G474D substitution. In addition to our in vivo and in vitro results, both Gly474 and Cys554 are highly conserved among bifunctional AK/HSDH enzymes in many organisms, suggesting that these residues are important for the HSDH activity of ThrA. Therefore, further biochemical and structural analysis may clarify the mechanism of the dysfunction caused by the G474D and C554Y substitutions.

In conclusion, intracellular lysine conferred high-temperature stress tolerance to *E. coli* cells. The lysine-mediated stress tolerance was accomplished by the enhancement of cellular lysine productivity and by lysine supplementation. Our findings suggest that microorganisms which overproduce lysine have the potential to enhance cellular tolerance to environmental stresses and could be applied as host strains for the microbial production of useful compounds due to their robust properties, leading to a decrease in cost for maintaining cultivation conditions.

## Supplementary Information

Below is the link to the electronic supplementary material.

Supplementary file1 (PDF 333 KB)

## Data Availability

The data underlying this article are available in the article.
