# Supplementary material for: Enhancement of lysine biosynthesis confers high-temperature stress tolerance to Escherichia coli cells
Source: Appl Microbiol Biotechnol. 2021 Aug 29;105(18):6899–908. doi: 10.1007/s00253-021-11519-0 (PMC8426250; doi:10.1007/s00253-021-11519-0)
Supplement: Supplementary file 1 — Supplementary file1 (PDF 333 KB) [file 253_2021_11519_MOESM1_ESM.pdf]

Applied Microbiology and Biotechnology

Enhancement of lysine biosynthesis confers high-temperature stress tolerance on *Escherichia coli* cells

Shota Isogai and Hiroshi Takagi*

Division of Biological Science, Nara Institute of Science and Technology, 8916-5 Takayama-cho, Ikoma, Nara 630-0192, Japan

*Corresponding author: Hiroshi Takagi

Email:hiro@bs.naist.jp, Tel: +81-743-72-5420, Fax: +81-743-72-5429

This file includes Fig. S1.

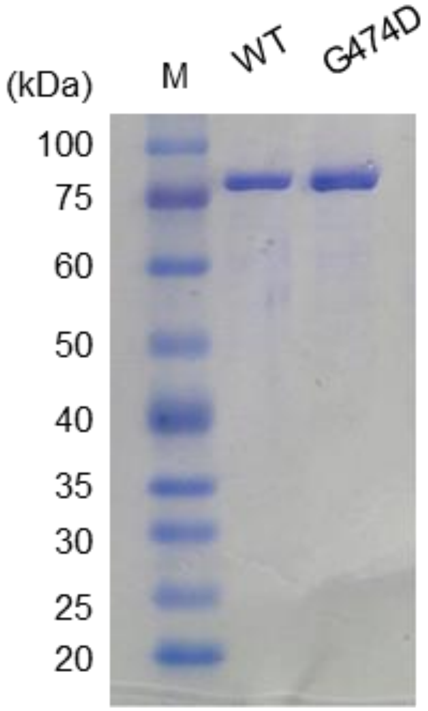

Fig. S1. SDS-polyacrylamide gel electrophoresis of the recombinant ThrA purified from *E. coli* BL21 (DE3) cells.

Lane M: Molecular mass standards, Lane WT: the wild-type ThrA, Lane G474D: the G474D variant ThrA
